# Mechanical Properties and Thermal Degradation Behaviour of Polyurethane Composites Incorporating Waste-Glass Particles

**DOI:** 10.3390/polym17131734

**Published:** 2025-06-21

**Authors:** Nathaphon Buddhacosa, Edwin Baez, Thevega Thevakumar, Everson Kandare, Dilan Robert

**Affiliations:** 1 Department of Civil and Infrastructure Engineering, School of Engineering, RMIT University, Melbourne, VIC 3000, Australia; junior.buddhacosa@rmit.edu.au (N.B.); ubernel@gmail.com (E.B.); s3915341@student.rmit.edu.au (T.T.); 2 Department of Civil Engineering, Faculty of Engineering, University of Peradeniya, Kandy 20000, Sri Lanka; 3 Department of Aerospace Engineering, School of Engineering, RMIT University, Melbourne, VIC 3000, Australia; everson.kandare@rmit.edu.au

**Keywords:** construction and building, glass/PU composites, mechanical properties, optimised manufacturing process, upcycling glass waste

## Abstract

This study investigated the effect of hot-pressing conditions, including the curing temperature, curing time and the applied pressure, on the flexural properties of polyurethane (PU) composites incorporating 88 wt.% (Glass/PU-88/12) and 95 wt.% (Glass/PU-95/5) recycled glass particles. Hot-pressing (cure) temperatures between 100 °C and 180 °C were investigated with the objective to shorten the cure cycle, thereby increasing the production rate of the glass/PU composites to match industrial scales. The hot-pressing time varied between 1 min and 30 min, while the pressure varied between 1.1 MPa and 6.6 MPa. Further to investigating the hot-pressing conditions, the effect of post-curing on the flexural properties of glass/PU composites was also investigated. Microstructural analysis was used to identify the interactions between the glass particles and the PU matrix, explore the void content and establish the relationship between the microstructure and the mechanical properties of the resultant glass/PU composites. Glass/PU composites incorporating 5 wt.% (Glass/PU-95/5), 10 wt.% (Glass/PU-90/10) and 12 wt.% (Glass/PU-88/12) were manufactured under optimised hot-pressing conditions (temperature = 100 °C; cure time = 1 min; pressure = 6.6 MPa) and evaluated under flexural, tensile and compression loadings. Furthermore, the high-temperature stability of the composites was evaluated using thermogravimetric analysis. This study demonstrates the feasibility of upcycling glass waste into value-added materials for potential use in the construction and building industry.

## 1. Introduction

The rapidly increasing global population and the prevalence of a disposable economy have led to the production of substantial volumes of waste in developed countries. Among the critical challenges posed by the surge in disposable products are effective waste management processes and technologies. Within this context, the recovery and reuse of discarded glass packaging cullets have emerged as areas facing significant hurdles. In particular, the lack of a seamless transition from recovery and manufacturing to end-use applications complicates the recycling process for glass waste. Other challenges and considerations hindering the uptake of glass waste into the mainstream economy include the lack of effective sorting and cleaning processes, incompatibility with the intended applications, insufficient market demand, an incomplete recycling loop and issues with logistics and transportation [1]. In Australia alone, over 1 million tonnes of glass are produced annually, yet only 33% of this glass is recycled, underscoring the immense untapped potential for resource recovery [2]. To support a circular economy and resource-efficient future, where materials are recycled and repurposed rather than discarded, the utilisation of recycled glass has gained prominence [1,3,4]. Recycled glass offers a valuable opportunity to enhance sustainability and add value to waste materials, especially in the high-volume construction and building sector [3,4,5].

Recycled glass has found application in the construction industry, including as a replacement for sand aggregate in concrete, as a substitute for cement in asphalt pavement and road infrastructure, including pit lids or access covers [5,6,7,8,9,10,11,12,13,14]. The diverse and high-volume applications of recycled glass in the construction industry highlight the versatility and environmental benefits of virgin materials including resource conservation, energy savings, waste diversion and reduced carbon footprint [3]. To increase confidence in products incorporating recycled glass and consequently enhance the appeal to manufacturing industries and end users in the construction sector, research and development efforts have concentrated on refining the design and manufacturing processes of such products [1,3]. Additionally, considerable attention has been devoted to developing energy-efficient cleaning methods for recovered glass waste and improving compatibility between glass particles and binders [15,16,17]. Furthermore, research has focused on optimising manufacturing processes and establishing the relationship between the microstructure and mechanical performance of waste glass composites for diverse applications [15,16,18,19].

Heriyanto et al. [15], developed polymeric glass composite (PGC) panels by incorporating 80–90 wt.% waste glass powder (particle size ranging between 64 µm and 6 mm) into an epoxy matrix. Increasing the content of untreated glass from 80 wt.% to 90 wt.% reduced the flexural and compressive strength of the composite by 39% (101 MPa to 62 MPa) and 15% (26 MPa to 22 MPa), respectively. The reduction in mechanical strength was attributed to poor glass/epoxy adhesion. In a related study, Heriyanto et al. [16] reported enhanced fire resistance of PGCs incorporating 85 wt.% glass particles. The PGCs exhibited comparable fire resistance properties to commercial natural stones like marble and granite, which lack polymeric binders. These findings are particularly promising, especially when considering the potential application of glass-reinforced polymer composites in fire-prone infrastructure.

Sadik et al. [19] developed a composite utilising approximately 30 µm sized waste soda-lime glass powder (up to 30 wt.%) and recycled high-density polyethylene (RHDPE) through compression moulding. In comparison to the neat RHDPE, the addition of glass powder at 30 wt.% resulted in a 42% and 19% reduction in the tensile strength and in Young’s modulus, respectively. The decline in tensile properties was attributed to poor adhesion between the polymer matrix and the glass particles, leading to agglomeration. Aggregated glass particles are less effective in facilitating stress transfer, consequently leading to crack initiation [19]. Moreover, for relatively high glass content, the elongation at break decreased by as much as 98%, underscoring the increased brittleness with an increased volume fraction of glass fillers.

Research conducted in our laboratories by Robert et al. [18] investigated the effect of cure time on the flexural and compression properties of polyurethane (PU) matrix composites incorporating glass particles, inorganic fillers and pigments. In that research, the glass content varied between 55 wt.% and 65 wt.%, while the PU content was fixed at 28 wt.%. Generally, the flexural properties increased with extended cure time. Specifically, for a composite incorporating 62 wt.% of the 200–300 µm glass particles (F2-PS3), the flexural strength increased by as much as 16 times (1.1 MPa to 18.5 MPa) when the cure time increased from 14 days to 28 days. Similarly, the flexural modulus of F2-PS3 increased by more than 12-fold (0.12 GPa to 1.52 GPa) upon extending the cure cycle from 14 days to 28 days. This increase in the flexural properties was attributed to enhanced crosslinking because of the extended cure cycle. Despite the substantial polymeric binder content of 28 wt.%, glass/PU composites produced following the 14-day cure cycle did not achieve adequate mechanical properties to meet design load requirements for load-bearing structures. The mechanical properties required for load-bearing structures can only be achieved when the glass/PU composite undergoes ambient temperature curing over an extended period. However, in real-world, industry-scale applications, it is crucial that production processes are expedited to reduce manufacturing costs and meet product demand. Consequently, it is essential to identify rapid manufacturing conditions for the glass/PU composites that achieve acceptable mechanical properties and minimise production time and costs.

Employing elevated cure temperatures and post-curing processes can significantly reduce manufacturing time while still achieving optimal mechanical performance. In this study, we have explored curing temperatures that are substantially higher than ambient conditions with the objective to shorten manufacturing times. In addition to hot pressing and curing the glass/PU composites at elevated temperatures ranging between 100 °C and 180 °C, this research work also investigated the effect of post-curing on the mechanical properties of the glass/PU composites. A combination of elevated cure temperatures and post-curing can shorten the manufacturing time while achieving optimal mechanical performance. The glass/PU composites hold promise for use in fire-threatened infrastructure and meeting stringent fire safety standards necessitates minimising the flammable component, typically the polymeric binder. However, reducing the polymeric binder content to meet these safety requirements can potentially compromise the mechanical performance of the composites. To address this challenge, this study investigates the simultaneous application of pressure ranging between 1.1 MPa and 6.6 MPa during the manufacturing process. This approach aims to enhance composite consolidation and reduce void content, particularly in formulations with low polymer binder content.

It is critical to understand the relationship between the microstructure and the mechanical properties of the newly developed glass/PU composites. In this study, X-ray computed tomography (CT) and scanning electron microscopy (SEM) were used to examine the interaction between the glass particles and the polymeric binder, as well as the void content and the interfacial bonding integrity between constituent elements of the glass/PU composite. Before the glass/PU composites can be confidently adopted in fire-prone applications, it is imperative that materials scientists and engineers are confident the new composite will meet the stringent fire safety standards, especially in the building and construction industry. The thermal degradation behaviour of glass/PU composites was therefore evaluated using thermogravimetric analysis. This study aims to develop sustainable building materials using recycled glass, fostering a transition towards eco-friendly construction materials.

## 2. Materials and Methods

### 2.1. Materials

The PU matrix used to bind together glass particles in the composite material was synthesised via a polymerisation reaction between two polymeric precursors: polyethylene glycol (polyol) supplied by Redox, Minto, Australia and a diphenylmethane diisocyanate (MDI) curing agent supplied by Brenntag, Australia. These precursors were used without further modification and following the recommended isocyanate-to-polyol mixing ratio of 3:2. Contaminant-free glass particles recovered from mechanical crushing of recycled glass collected from metropolitan Melbourne were supplied by RepurposeIt, Epping, Australia. The average particle size of the supplied glass fines was further reduced by acoustic grinding. ASTM-certified sieves were employed to separate the glass particles into two distinct granulometric fractions: particles with sizes below 300 μm and those exceeding 300 μm. Previous investigations demonstrated that the incorporation of glass particles with dimensions below 300 μm into a polymeric binder results in composite materials exhibiting high mechanical properties [18]. The particle size distribution (PSD) and dimensions of the sieved glass fines were characterised using a Malvern Mastersizer 3000 apparatus (Malvern Panalytical, Malvern, UK).

### 2.2. Glass/PU Composite Fabrication

**Figure 1:**
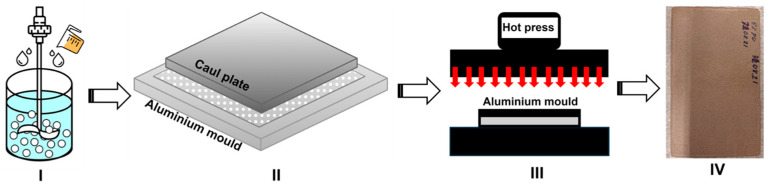
Schematic of the glass/PU hot-pressing manufacturing process: (**I**) mixing glass particles and PU, (**II**) transfer to mould, (**III**) hot pressing and (**IV**) demoulded composite.

Glass particles that passed through the sieve were dried at 110 °C for at least 12 h—until consecutive measurements using a 0.0001 g-precision balance showed no further mass change—before blending with the appropriate amounts of polyol and cyanate components. Complete drying of the glass particles is critical, as even trace moisture can react with isocyanate and compromise the integrity of the glass/PU composite. The glass/PU composite was manufactured following the processes described below. The first formulation consisted of 95 wt.% glass particles mixed with 5 wt.% PU (Glass/PU-95/5), while the second formulation involved 88 wt.% glass particles mixed with 12 wt.% PU (Glass/PU-88/12). The glass/PU mixture was homogenised using a mechanical stirrer operating at 600 rpm for 5 min. Subsequently, the resultant mixture was poured into a Teflon^®^ film-lined steel mould and covered with a thick steel caul plate. The glass/PU mixture was then hot pressed at varying temperatures (100–180 °C), times (1–30 min) and pressures (1.1–6.6 MPa) using a hydraulic-operated hot press. A schematic of the manufacturing conditions for glass/PU composites is shown in Figure 1. The hot-press conditions (pressure, temperature and time) were each varied systematically—one parameter at a time—while the other two were held constant (see Table 1). The flexural properties of the resulting glass–PU composites were then evaluated. The composite cure temperature of 160 °C and time of 1 min produced the highest flexural performance and was therefore designated the optimal hot-pressing manufacturing process. The neat PU was prepared by mixing the polyol and cyanate portions according to the manufacturer’s recommendations. The PU was allowed to cure at room temperature over a 24 h period before thermogravimetric experiments were conducted.

The curing conditions for the glass/PU composites were optimised based on the hot-pressing temperature, duration and pressure, as indicated in Table 1. The composites were conditioned at room temperature (23 °C) over a period of 24 h before conducting flexural tests. Some Glass/PU-95/5 composites, produced by hot pressing the glass/PU mixture for 1 min under an applied pressure of 6.6 MPa at temperatures ranging between 100 °C and 160 °C, underwent post-curing at 80 °C for 24 h. Following the optimisation of the glass/PU composite manufacturing conditions, the effect of the polymer binder content (5 wt.%, 10 wt.% and 12 wt.%) on the flexural, tensile and compression properties of the glass/PU composites was also evaluated, as indicated in Table 2.

### 2.3. Evaluating the Mechanical Properties of Glass/PU Composites

The glass/PU composite manufacturing conditions were optimised based on the flexural properties measured in accordance with the ASTM D2344-22 standard [20]. The test specimens measuring 100 mm× 20 mm× 12 mm were subjected to three-point bending using a 50 kN Instron 5900R Universal (Instron, Norwood, MA, USA) testing machine operated at a crosshead displacement rate of 1 mm/min. The loading was stopped once the specimens failed. At least five flexural specimens were tested for each glass/PU composite configuration, from which the average flexural properties and the corresponding standard deviation values were calculated. Tensile tests were performed in accordance with the ASTM D638-22 standard [21]. Rectangular-shaped specimens measuring 170 mm× 20 mm× 9 mm were subjected to tensile loading using the Zwick/Roell Universal testing machine operated at a crosshead displacement rate of 1 mm/min until failure. At least five specimens were tested for each glass/PU composite configuration, and the average tensile properties with their respective standard deviation were reported. Only the test specimens that failed outside the grip area (i.e., within the gauge section) were considered in computing the average and the standard deviation.

Compression properties were evaluated using rectangular specimens with dimensions of 12.7 mm× 12.7 mm× 25.4 mm using a 50 kN Instron 5569 Universal (Instron, Norwood, MA, USA) testing machine in accordance with the ASTM D695 standard [22]. The compression load was applied at a crosshead displacement rate of 1 mm/min until failure. At least five specimens for each glass/PU composite configuration were tested. The average compression properties were reported together with the corresponding standard deviation.

### 2.4. Microstructural Analysis of Glass/PU Composites

SEM analysis was conducted to investigate the influence of PU binder content and the manufacturing conditions on the interaction between glass particles and the PU matrix and consequently the glass/PU composite microstructure. Prior to image acquisition, the glass/PU composites were cast in a cold-curing polymer matrix using 25 mm diameter Fixiform mounting tubes (Struers Australia, Milton, Australia). The glass/PU composite specimens were polished to a smoothness of 1 µm using the Struers Tegramin-25 polisher (Struers Australia, Milton, Australia). Following polishing, a thin layer of iridium was sputter coated onto the specimens using the Leica EM ACE600 sputter (Leica, Wetzlar, Germany) coater operated at a pressure of 8 × 10^−3^ mbar, resulting in a coating thickness of approximately 5 nm. SEM imaging was conducted using the FEI Quanta 200 SEM (FEI, Hillsboro, OR, USA) operated at an accelerating voltage of 5 kV, spot sizes ranging from 3 to 5 and magnifications of 200× and 2000×. Microscopic analysis of the glass/PU composites allowed for detailed characterisation and analysis of the microstructure and the interactions between constituent elements. The microstructural characterisation is pivotal for establishing the relationship between the microstructure and mechanical properties of the glass/PU composites. This understanding is essential for elucidating the underlying mechanisms governing the mechanical response of glass/PU composites and can ultimately inform strategies for optimising mechanical performance.

The influence of the polymeric binder content and manufacturing process on the porosity of the glass/PU composites was assessed using a General Electric Phoenix v|tome|xs X-ray CT (Waygate Technologies, Hürth, Germany). The CT characterisation aimed to quantify the porosity volume fraction and visualise the internal microstructure of the glass/PU composites to augment the 2D surface images acquired using the SEM. The CT scans were acquired at 80 kV and 100 µA, with a voxel length of 20 µm. During the scanning process, up to 2000 2D image projections were acquired and subsequently reconstructed into 3D volumetric image projections using the VolMaxStudio 3.0 software. The Porosity Analysis function was then utilised to determine the void volume fraction within partitioned regions of the 3D volumetric image projections. The “Only Threshold” algorithm with an interpolation factor of 0.50 was applied to segment void space from solid material. Once a global grey-value threshold was chosen (based on the histogram valley between solid and air), the software positioned the material–void interface halfway between adjacent voxels that straddled that threshold. To ensure reproducibility, the same threshold and interpolation settings were applied to all composite scans, and the resulting iso surface was visually overlaid on raw slices to confirm accurate pore delineation. While voids with sub-threshold sizes (e.g., pores smaller than ~40 µm (i.e., two voxels)) were underrepresented or entirely omitted from the analysis, the relative ranking of porosity across different composite samples was still valid. Understanding the relationship between void content, manufacturing conditions and the polymeric binder content is crucial for optimising the fabrication process and enhancing the overall mechanical performance of the glass/PU composites.

### 2.5. Thermogravimetric Analysis (TGA) of Glass/PU Composites

The thermal stability of glass/PU composites was evaluated using a Perkin Elmer (STA 6000) instrument (Perkin Elmer, Waltham, MA, USA). The analysis was performed using 10–15 mg samples of the glass/PU composites under flowing air conditions (at a rate of 20 mL/min), with a heating rate of 20 °C/min between 35 °C and 800 °C. The TGA experiments aimed to elucidate the thermal stability and decomposition behaviour of the glass/PU composites, providing valuable insights into their performance under temperature conditions simulating real-world exposure to high-intensity heat flux or fire. While inert-atmosphere TGA can isolate pyrolysis pathways, this study was focused on thermo-oxidative stability; future work may compare inert and oxidative data to further elucidate pure pyrolysis mechanisms. Comparing the thermal behaviour of glass/PU composites with varied compositions under air was sufficient to meet the objectives of this investigation.

## 3. Results and Discussion

### 3.1. Optimisation of the Manufacturing Process

**Figure 2:**
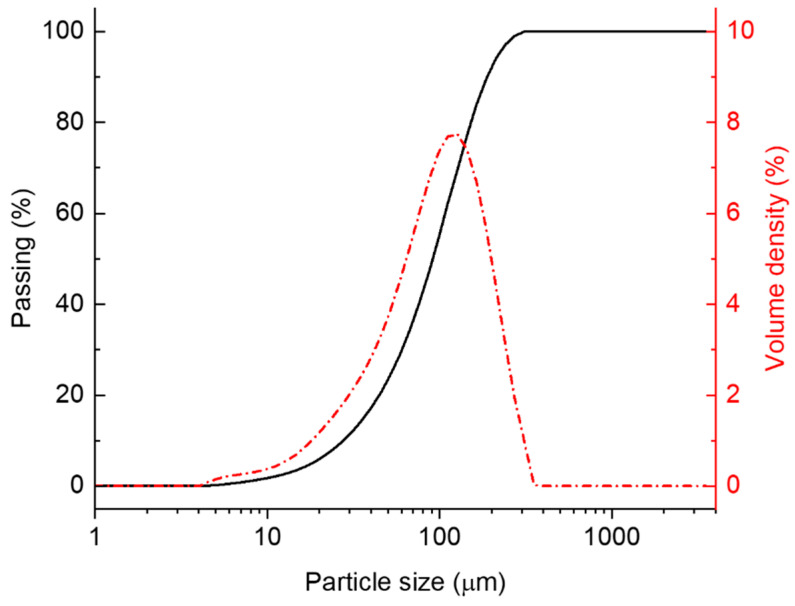
Particle size distribution (PSD) of glass particles following acoustic grinding showing the passing (%) and volume density (%) functions.

The size distribution of glass particles, obtained through acoustic grinding and which passed through a 300 µm mesh sieve, is shown in Figure 2. The passing (%) and volume density plots indicate that more than 98% of the glass particles that passed through the sieve had an average particle size below 300 µm. The nominal reported size of the glass particles was 110 µm, and their dimensions ranged between 5 µm and 350 µm. Although a 300 µm aperture sieve was used for glass particle classification, some elongated particles with one of the dimensions greater than 300 µm passed through the sieves when aligned vertically. As a result, the volume density distribution shows a small tail up to ~350 µm, representing a small fraction of glass particles whose longest dimension exceeds the sieve aperture. This study assessed the impact of various manufacturing conditions on the flexural properties of glass/PU composites including the hot-pressing temperature, duration, pressure and post-curing effects. The goal was to identify critical parameters that influence the flexural properties of the resulting glass/PU composites and determine optimal manufacturing conditions for high-volume production. Firstly, the study examined the effect of hot-pressing temperature on the flexural properties of the Glass/PU-95/5 composites between 100 °C and 180 °C. Hot-pressing pressure and duration were maintained at 6.6 MPa and 1 min, respectively. The PU content was limited to 5 wt.% to prevent runaway thermal reactions during elevated temperature curing. It is well established that the crosslinking density of polymers, including PU, is directly influenced by the curing temperature, with higher curing temperatures resulting in increased crosslinking density [23]. While elevated crosslinking density has been shown to enhance the glass transition temperature and, consequently, the mechanical properties of the PU matrix [24], it also reduces the strain to failure, increasing the material brittleness [25].

**Figure 3:**
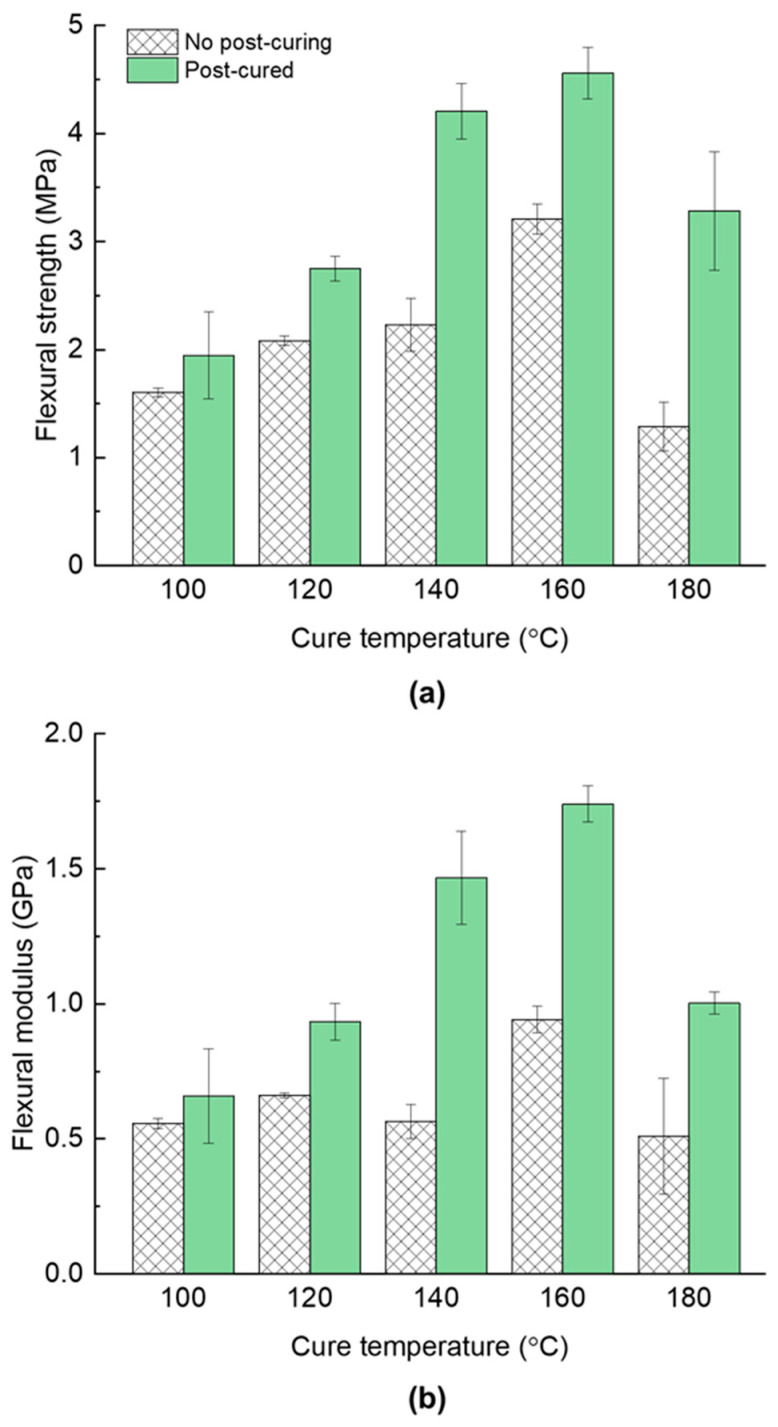
The influence of hot-pressing temperatures between 100 °C and 180 °C, and the effect of 80 °C, 24 h post-curing on the flexural (**a**) strength and (**b**) modulus of PU/glass-95/5 composites manufactured under an applied pressure of 6.6 MPa over 1 min.

As shown in Figure 3, the flexural strength of the Glass/PU-95/5 composite generally increased with the increase in hot-pressing (cure) temperatures, beginning at 1.7 MPa at 100 °C and peaking at 160 °C (3.3 MPa), before decreasing to 1.2 MPa at 180 °C. The flexural modulus displayed a similar trend, beginning at 0.5 GPa at 100 °C, reaching 1 GPa at 160 °C and then decreasing to 0.5 GPa at 180 °C. The improvement in the flexural properties of the Glass/PU-95/5 composite, as the cure temperature increased, can be attributed to the augmented crosslinking of the thermosetting PU polymer [23,24]. However, it is essential to recognise that beyond a specific temperature threshold, high crosslinking densities may yield diminishing returns due to the elevated brittleness of the PU matrix [25]. In this study, this threshold temperature is 160 °C. At higher temperatures, the PU matrix becomes highly crosslinked, resulting in an overly rigid and brittle composite with reduced strain to failure [26,27,28]. The decline in flexural strength may also reflect early-stage thermal degradation and chain scission of the PU network. Prolonged exposure at elevated temperatures can trigger thermo-oxidative cleavage of urethane linkages, lowering the molecular weight of the matrix hence the mechanical performance.

The effect of post-curing (e.g., exposure at 80 °C over 24 h) on the flexural strength and modulus of the Glass/PU-95/5 composite panels was investigated and the results are presented in Figure 3. The post-cured specimens exhibited a significant improvement in both the flexural strength and modulus compared to the non-post-cured composites. For instance, post-curing the Glass/PU-95/5 composite fabricated at 160 °C resulted in a 42% increase in the flexural strength (3.2 MPa to 4.6 MPa) and an 85% improvement in the flexural modulus (0.9 GPa to 1.7 GPa) compared to the composite, which was not post-cured. These results suggest that after the initial curing of the panels, further crosslinking continues to occur in the polymeric matrix during the post-curing process. A post-cure treatment at 80 °C occurs well below the onset of significant polymer breakdown, enabling network relaxation and secondary crosslinking without thermal degradation—thereby improving the flexural performance.

Given the limited moulding time (60 s), the relatively low thermal conductivity of the glass/PU composite and the one-dimensional (1D) heat transfer from the heated platens into the glass/PU composite system, it is more likely that the core temperature of the composite panel did not reach the target curing temperature. Only the sections of the glass/PU composite panels closest to the heated platens achieved the target curing temperature. Consequently, the crosslinking density within the core of the composite panels was relatively lower than the crosslinking density closer to the heat-exposed composite surfaces. The additional thermal energy during post-curing thus increased the crosslinking density within the core of the composite panels, subsequently enhancing the flexural properties. Although there were diminishing returns regarding the flexural properties at curing temperatures above 160 °C, post-curing composite panels manufactured at 180 °C still resulted in an increase in the flexural properties. Despite subjecting the composite panel system to a relatively high curing temperature of 180 °C, the heat transfer into the composite core was still limited to a 1D flow. Therefore, post-curing composites hot pressed at 180 °C provided an opportunity for further crosslinking within the composite core section consequently improving the flexural properties of the glass/PU composites (see Figure 3).

**Figure 4:**
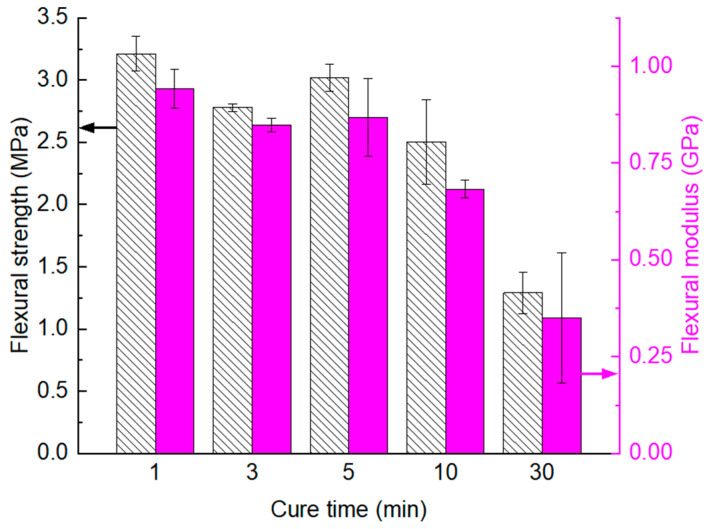
Influence of hot-pressing time on the flexural strength (pattern fill) and modulus (solid fill) of the non-post-cured Glass/PU-95/5 composite hot pressed at 160 °C under a pressure of 6.6 MPa with curing times ranging between 1 min and 30 min.

Having established the optimal curing temperature as 160 °C, the next step was to examine the influence of cure time (hot-pressing time) on the flexural properties of glass/PU composites. The curing time was varied between 1 min and 30 min (e.g., 1 min, 5 min, 10 min and 30 min) with the resultant flexural properties shown in Figure 4. The variations in the flexural strength and modulus of the Glass/PU-95/5 composite for curing times ranging from 1 min to 5 min were statistically insignificant. However, beyond the five-minute curing period, both the flexural strength and modulus significantly decreased with the increase in hot-pressing time.

For example, when compared to the one-minute hot-press cycle, the composites cured for 10 min and 30 min, respectively, exhibited a 22% and 60% reduction in flexural strength, as well as a 27% and 63% decrease in flexural modulus. The decrease in flexural properties for prolonged curing times (e.g., 10 min to 30 min) was attributed to excessive crosslinking within the polymer matrix closest to the heated platens. While relatively high crosslinking density increases the flexural strength and modulus, it can, however, have detrimental effects on the fracture toughness of the composite material [29]. Therefore, prolonged exposure of the Glass/PU-95/5 composite to 160 °C reduced the strain to failure, consequently lowering the flexural properties. Given the statistically insignificant variations in the flexural properties of the Glass/PU-95/5 composites cured between 1 min and 5 min, the shortest curing time of 1 min was selected for further work as it would translate into reduced manufacturing time and costs, especially at the industrial scale.

**Figure 5:**
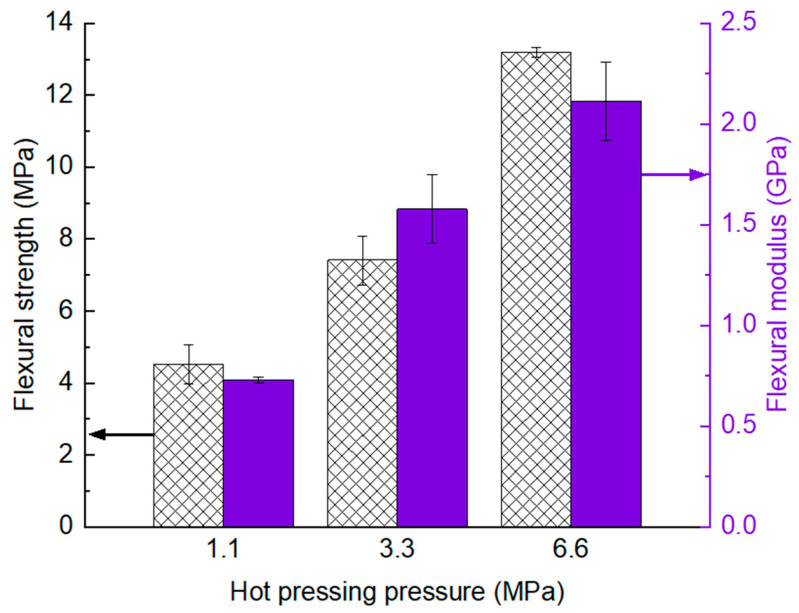
Effect of hot-pressing pressure on the flexural strength (pattern fill) and modulus (solid fill) of the non-post-cured Glass/PU-88/12 composite hot pressed at a temperature of 160 °C for 1 min.

Since we did not encounter notable issues related to runaway exothermic reactions when curing the Glass/PU-95/5 composite even at 180 °C, the PU content was increased from 5 wt.% to 12 wt.% with respect to the glass/PU composite panel weight. Consequently, the effects of hot-pressing pressure on the flexural properties of the glass/PU composites were investigated using the Glass/PU-88/12 composite. During hot pressing, the applied pressure was varied between 1.1 MPa and 6.6 MPa, as indicated in Table 1. Figure 5 shows the flexural properties of Glass/PU-88/12 composites manufactured using three different hot-pressing pressures of 1.1 MPa, 3.3 MPa or 6.6 MPa. The results clearly indicate that increasing the hot-pressing pressure enhances the flexural properties of the resulting glass/PU composite. Specifically, increasing the hot-pressing pressure from 1.1 MPa to 6.6 MPa improved both the flexural strength and modulus of the Glass/PU-88/12 composite by 192% and 190%, respectively.

Relatively high moulding pressures improve consolidation of the glass/PU composites, minimising internal voids and enhancing glass particle–matrix interfacial strength. Previous research in our labs on a comparable glass/epoxy composite with a 12 wt.% epoxy matrix showed that raising the moulding pressure from 1.1 MPa to 6.6 MPa reduced the void volume fraction from 11 vol % to 1 vol % [30]. Correspondingly, the density of the glass/epoxy composite increased from 1.67 g/cm^3^ at 1.1 MPa to 1.84 g/cm^3^ at 6.6 MPa, indicating an about 10% improvement in compaction, aligning with the 11% reduction in void content. In that same study, increasing the moulding pressure from 1.1 MPa to 6.6 MPa enhanced the flexural strength of the 12 wt.% epoxy glass/epoxy composite by 67% (from 15 MPa to 25 MPa) and the flexural modulus by 45% (from 3.8 GPa to 5.5 GPa). These gains in flexural performance were ascribed to better composite consolidation and a reduction in internal voids, which otherwise would have compromised the mechanical properties of the composite [16]. Polymeric binder starvation can also lead to disbonded glass particle–matrix interfaces, which can contribute to reduced mechanical properties [15,16].

The investigation into the effects of hot-pressing temperature, time and pressure established the optimal processing conditions for the Glass/PU-88/12 composite system to be 160 °C, a pressing time of 1 min and a hot-pressing pressure of 6.6 MPa. This manufacturing process optimisation study also underscored the significance of post-curing (80 °C over 24 h), which enhanced the crosslinking density and subsequently improved the flexural properties [24]. With the manufacturing process optimised, the study then focused on examining the effect of polymer content on the thermal stability and selected mechanical properties (e.g., flexural, tensile and compression) of the glass/PU composite systems.

### 3.2. Effect of Binder Content on the Mechanical Properties of Glass/PU Composites

**Figure 6:**
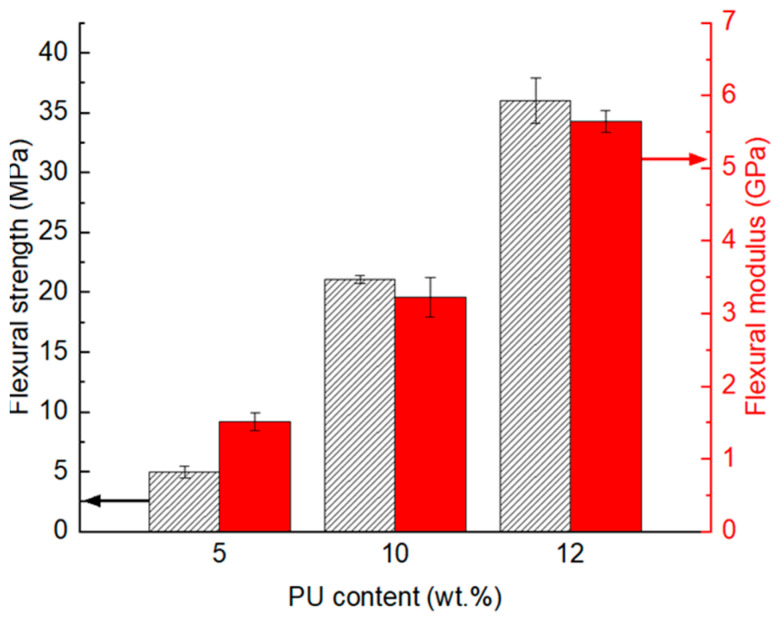
Flexural strength (pattern fill) and modulus (solid fill) of the glass/PU composites incorporating 5 wt.% (Glass/PU-95/5), 10 wt.% (Glass/PU-9/10) and 12 wt.% (Glass/PU-88/12) polymeric binder. The glass/PU composites were hot pressed at 160 °C, under a pressure of 6.6. MPa for 1 min followed by post-curing at 80 °C over 24 h.

Glass/PU composites with a polymeric binder content of 5 wt.%, 10 wt.% or 12 wt.% were manufactured using optimised hot-pressing conditions (e.g., cure temperature of 160 °C, 6.6 MPa applied pressure, 1 min hot pressing). These glass/PU composites with different binder content were post-cured at 80 °C for 24 h before being subjected to flexural, tensile and compression tests. Figure 6 shows the flexural properties of the glass/PU composites incorporating 5 wt.%, 10 wt.%, or 12 wt.% PU. Increasing the polymeric binder content increased the flexural strength of the resulting glass/PU composites. Flexural strength values of 5 MPa, 21 MPa and 36 MPa were measured for glass/PU composites incorporating 5 wt.%, 10 wt.% and 12 wt.% PU, respectively. Similarly, the flexural modulus increased with the increase in the PU content. Flexural moduli values of 1.5 MPa, 3.2 MPa and 5.7 MPa were measured for glass/PU composites, respectively, incorporating 5 wt.%, 10 wt.% and 12 wt.% PU. The enhancement in flexural properties with the increase in the PU content can be attributed to two factors. Firstly, the increase in the polymeric binder reduces porosity in the resulting glass/PU composites, as the polymer infiltrates the pores between the glass particles. Secondly, the higher polymeric binder content enhances interfacial bonding between the glass particles and the polymer matrix, resulting in increased adhesion and flexural load-carrying capacity. These outcomes are consistent with findings reported elsewhere in the literature [15,16,19].

**Figure 7:**
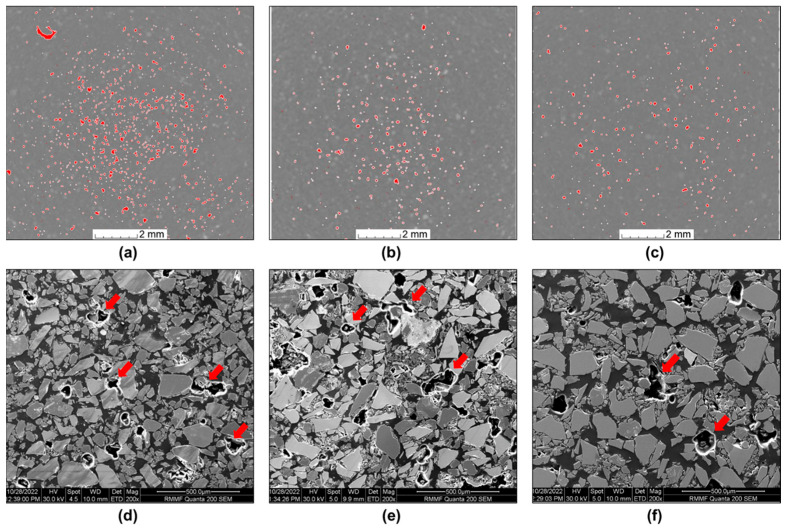
CT images of (**a**) Glass/PU-95/5, (**b**) Glass/PU-90/10 and (**c**) Glass/PU-88/12 composite, respectively. SEM images of (**d**) Glass/PU-95/5, (**e**) Glass/PU-90/10 and (**f**) Glass/PU-88/12 composites, respectively. The red speckles in the CT images indicate the voids, while porosity in SEM images is indicated by red arrows in (**d**–**f**).

**Figure 8:**
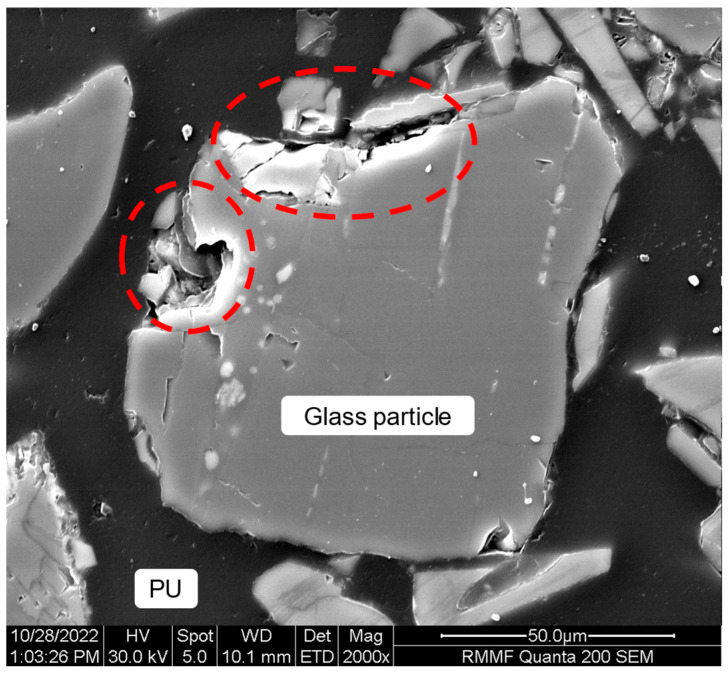
SEM image of the Glass/PU-95/5 composite revealing debonding at the polymeric binder and glass particle interface (within red dashed lines).

The influence of the polymeric binder content on the void content in the glass/PU composites is revealed in Figure 7. The CT images (Figure 7a–c) show that increasing polymeric content resulted in reduced porosity, with the void volume fraction decreasing from 2.7% to 1% as the PU content increased from 5 wt.% to 12 wt.%. It is noteworthy that the modest reduction in CT-measured porosity from 2.7 vol.% to 1.0 vol.% (Figure 7) contrasts sharply with the ~7-fold increase in flexural strength (Figure 6). Thus, it is important to note that CT analyses only captured voids with a voxel size of at least ~40 µm (i.e., two adjacent voxels), with smaller interfacial gaps and microcracks remaining undetected. The resolution limit was therefore more pertinent for the 12 wt.% PU composite, where the true void content was relatively low, and a potentially significant fraction of sub-voxel pores remained undetected. This is a possible limitation of relying on CT images, as they may not capture disbonding cracks, which, although small, can still have a substantial adverse impact on the composite’s mechanical performance. Indeed, Figure 8 shows debonding at the glass particle–PU interface in the 5 wt.% PU composite, highlighting sub-resolution flaws that may possibly act as stress concentrators under flexural loading. By increasing PU content, not only is macroscopic porosity reduced, but microscopic interfacial defects are also effectively eliminated through improved wetting of the glass particles. The richer PU phase thus forms a continuous, highly crosslinked network that (1) enhances load transfer between the matrix and the reinforcement, (2) increases fracture toughness via superior crack-bridging and (3) fills microvoids below the CT detection threshold. Together, these improvements in interfacial integrity, stress transfer, matrix stiffness and defect mitigation can explain the dramatic enhancement in flexural strength with increasing PU content. Reduced porosity and improved interfacial bonding between glass particles and the PU matrix enhance the integrity of the composite, hence its flexural properties [31,32].

The CT images provide information at a macroscale level regarding the interactions between the glass particles and the polymeric matrix, while SEM analysis allows for microscale observations of the same interactions. The SEM images of the glass/PU composites incorporating varied PU content are shown together with the CT images in Figure 7. It is apparent that the void content in the glass/PU composites decreases with the increase in the PU content. The void content in Glass/PU-95/5 (Figure 7d) was significantly greater than the corresponding void volume fractions in the Glass/PU-90/10 (Figure 7e) and Glass/PU-88/12 (Figure 7f) composites.

Scanning electron microscopic analysis of the glass/PU composites unveiled debonding at the glass particle/PU matrix interface, as shown in Figure 8 for the Glass/PU-95/5 composite. Interfacial debonding was attributed to the mismatch in coefficients of thermal expansion (CTE) between the glass particle and PU matrix, which caused disbonding as the composite cooled down from the cure (160 °C) or post-cure (80 °C) temperature [33]. The literature-reported CTE values for glass particles (8.2–9.9 × 10^−6^ K^−1^) [34] are an order of magnitude lower than those reported for the PU matrix (124–164 × 10^−6^ K^−1^) [35], which can result in material separation. Additionally, relatively low volume fractions of the binder can promote interfacial debonding region between the matrix and the irregular glass particle surface, consequently resulting in lower mechanical properties for the resultant composites [33]. The CT and SEM microstructural analysis established a clear relationship between the particle–matrix interphase and the flexural properties. As the applied load is redistributed via the matrix during mechanical loading, the composite with low polymeric binder content (Glass/PU-95/5) cannot efficiently support stress transfer, leading to the initiation of cracks, which then propagate leading to failure. Sadik et al. [19] reported similar findings in which mechanical properties degraded due to reduced interfacial bonding between the polymeric matrix and the high-volume fraction waste-recovered glass fillers. Surface modification of glass particles may enhance their compatibility with the polymeric binder [15,16,36]. However, it should be noted that surface functionalisation of the glass particles can increase manufacturing cost and complexity at the industrial production scale. Therefore, the benefits of surface modification, with regard to improving mechanical properties, should be carefully weighed against these potential drawbacks.

**Figure 9:**
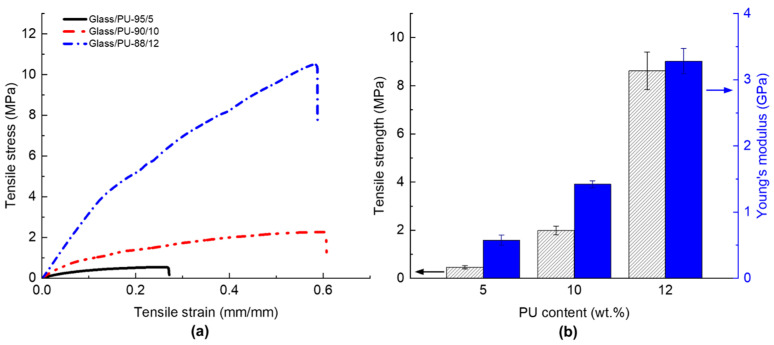
(**a**) The tensile stress–strain profiles and (**b**) the tensile strength (pattern fill) and modulus (solid fill) of glass/PU composites with PU content varying between 5 wt.% and 12 wt.%. The glass/PU composites were hot pressed at 160 °C, under a pressure of 6.6. MPa for 1 min followed by post-curing at 80 °C over 24 h.

The tensile behaviour of glass/PU composites incorporating 5 wt.% to 12 wt.% of the polymeric binder was investigated, and the tensile properties are shown in Figure 9. The tensile stress–strain profiles are shown in Figure 9a, while the extracted tensile strength and tensile modulus are presented in Figure 9b. Like the trend observed for flexural properties, an increase in PU content resulted in higher tensile strength and modulus. Specifically, the tensile strength increased from 0.5 MPa to 8.6 MPa when the PU content was increased from 5 wt.% to 12 wt.% (Figure 9b). The tensile modulus followed a similar trend, with a more than four-fold increase (0.4 GPa to 1.8 GPa) when the PU content increased from 5 wt.% to 12 wt.%. The improvement in tensile properties, akin to the flexural properties, was attributed to enhanced interfacial bonding between the glass particles and the PU matrix, as well as reduced void content due to the increased binder content. The tensile stress–strain profiles of the glass/PU composites with varied binder content (Figure 9a) revealed a substantial reduction in the strain to failure as the binder content was reduced. The composites with relatively low binder content have insufficient polymeric matrix to support stress transfer between the glass particles, ultimately resulting in brittle failure.

**Figure 10:**
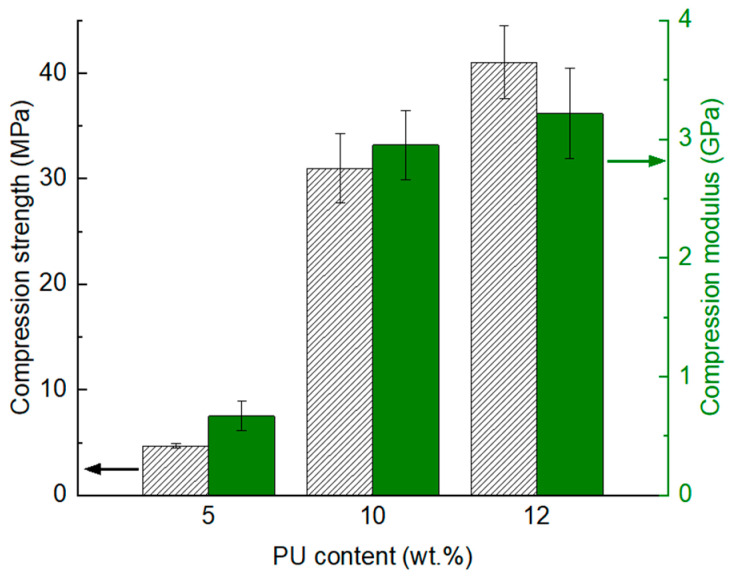
Compression strength (pattern fill) and modulus (solid fill) of the glass/PU composites incorporating a 5 wt.% (Glass/PU-95/5), 10 wt.% (Glass/PU-9/10) and 12 wt.% (Glass/PU-88/12) polymeric binder. The glass/PU composites were hot pressed at 160 °C, under a pressure of 6.6. MPa for 1 min followed by post-curing at 80 °C over 24 h.

Glass particle-reinforced polymer composites are more likely to be adopted in the building and construction sector where they are prone to undergo compression loading. Therefore, it is critical to understand the compression response of the glass/PU composites as well. The compression properties of glass/PU composites incorporating different polymeric binder content are shown in Figure 10. The increase in polymeric content from 5 wt.% to 12 wt.% improved the compression strength from 4.7 MPa to 41 MPa. The compression modulus also increased with the increase in the polymeric binder content. The compression modulus increased by more than four-fold from 0.7 GPa to 3.2 GPa as the PU content increased from 5 wt.% to 12 wt.%. The increase in the polymeric binder content improved interfacial adhesion between the glass particles and the matrix, thereby enhancing the compression properties. However, when the PU content was low (i.e., 5 wt.%), the cracks initiating under compression loading propagated rapidly through the composite due to the presence of voids within the matrix and disbonded glass/PU interfaces. In contrast, when the concentration of polymeric binder was relatively high (i.e., 12 wt.%), the improved interfacial bonding with the glass, which reduced the void content and debonded interfaces consequently slowed down crack propagation.

The relationship between flexural, tensile and compression properties with the polymeric binder content reveals an increase in mechanical properties with increasing binder content. Glass/PU composites with reduced polymer binder content generally have inferior mechanical properties. This observation has been attributed to the increased void content at relatively lower polymeric binder content, as well as debonding at the glass/matrix interface due to the limited adhesion. This research has demonstrated the feasibility of using recovered waste glass to create composites with mechanical properties that are comparable to those of materials generally used in the building and construction industry [13,33]. The research outcomes from this work revealed the potential for upcycling glass waste into value-added products that contribute to a circular economy. While the mechanical performance of recovered glass/PU composites is promising, some of these composites may end up in fire-prone applications. Therefore, it is important to develop an understanding of how these new composites will behave when subjected to high temperatures. Consequently, the focus of the research shifted to investigating the thermal stability of glass/PU composites via thermogravimetric analysis.

### 3.3. Thermal Degradation Behaviour of Glass/PU Composites

**Figure 11:**
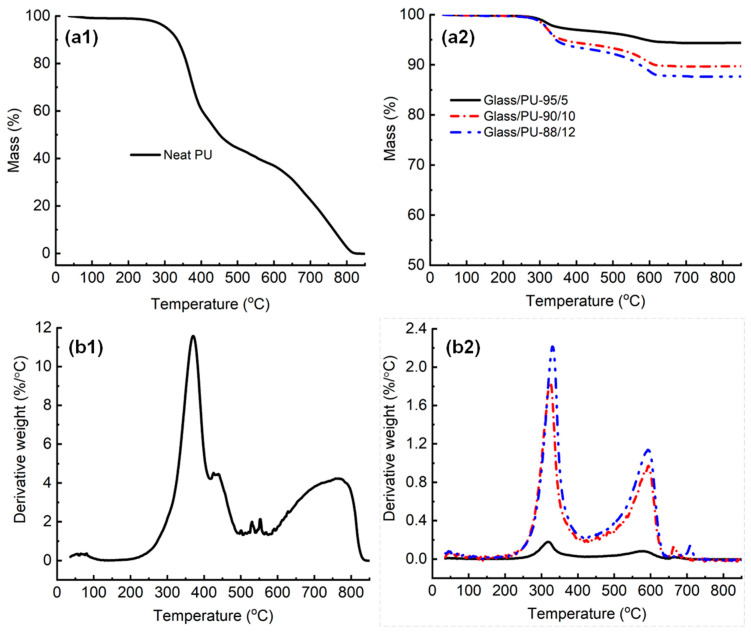
(**a1**,**a2**) The mass loss (%)–temperature and (**b1**,**b2**) derivative thermogravimetric (DTG) profiles of neat PU and glass/PU composites with different weight fractions of PU (5 wt.%, 10 wt.% and 12 wt.%).

Thermogravimetric analysis was used to investigate the decomposition behaviour of glass/PU composites with varying polymeric binder content. Figure 11(a1,a2,b1,b2) show the mass–temperature and dTG-temperature profiles of glass/PU composites incorporating 5 wt.%, 10 wt.% and 12 wt.% PU, and the neat PU without glass particles. The PU decomposition proceeds in three major stages (see Figure 11(a1). The first dTG peak appears around 370 °C and is attributed to the breakdown of urethane linkages, mainly involving the isocyanate segments. The second, broader peak between 400 °C and 500 °C reflects the degradation of polyol segments. A third distinct peak centred around 765 °C corresponds to the decomposition of primary residual char and other thermally stable components [37]. The multi-step profile in the dTG curve highlights the complex thermal degradation mechanism of PU leading to the release of large quantities of volatiles over a wide temperature range, underscoring the limited fire resistance of neat PU [37].

The residual yield measured at 850 °C for the glass/PU composites decreased with the increase in PU content (Figure 11(a1,a2)). The residual mass at 850 °C for Glass/PU-95/5, Glass/PU-90/10 and Glass/PU-88/12 was found to be 95%, 90% and 88%, respectively. The remaining mass at 850 °C corresponded to the glass particle weight fraction in the glass/PU composite. These findings confirm the thermal inertness of glass particles which are primarily composed of thermally stable silicon dioxide (SiO_2_). On the other hand, the neat PU completely decomposes into volatiles at 850 °C. The derivative weight profile, depicted as a function of temperature in Figure 11(b1,b2), reveals two main decomposition stages in the glass/PU composites. Stage 1 (250–400 °C) involves the primary decomposition of the polymeric binder, while stage 2 (400–650 °C) involves the decomposition of the primary char, with higher binder content resulting in greater mass losses during this stage. Compared to the neat PU, stage 1 of the thermal decomposition process occurs ~40 °C earlier in the glass/PU composites. The secondary decomposition (stage 2) occurs ~175 °C earlier, suggesting reduced thermal stability of the PU matrix when combined with glass particles. The disruption in polymer chain regularity and potentially lower crystallinity due to the presence of glass particles may be responsible for the inferior thermal stability of PU in glass/PU composites compared to neat PU [37].

As shown in Figure 11(a2), the primary and secondary decomposition peak positions for glass/PU composites remained unchanged as PU content increased, indicating a consistent thermal degradation mechanism regardless of binder concentration. However, higher PU content can lead to increased heat release rates, total heat release and potentially greater smoke and toxic gas emissions during flaming combustion. The rising dTG peak heights with binder content—from 5 wt.% to 12 wt.%—suggest the increased generation of both combustible and non-combustible volatiles. While increasing PU enhances mechanical performance, it inevitably reduces thermal stability and fire resistance. Future research should investigate the flammability and combustibility of glass/PU composites for fire-prone engineering applications.

## 4. Conclusions

This research aimed to identify optimal manufacturing conditions for PU matrix composites incorporating recovered glass waste. Elevated curing temperatures (100–180 °C) were explored with the objective to shorten the manufacturing time and thereby reduce manufacturing costs. Further, the effect of curing time and post-curing were investigated in relation to the flexural properties of the glass/PU composites. The effect of PU content on the mechanical (flexural, tensile and compression) properties of glass/PU composites manufactured using optimised manufacturing conditions was investigated. Finally, the thermal degradation behaviour of glass/PU composites incorporating varied PU content was also investigated. The outcomes from this research are summarised below:

- The optimum hot-pressing (cure) temperature for non-post-cured glass/PU composites was found to be 160 °C when the hot-pressing time and pressure were 1 min and 6.6 MPa, respectively.
- Cure times between 1 min and 5 min resulted in statistically insignificant differences in the flexural properties of non-post-cured glass/PU composites. Cure times greater than 5 min led to a reduction in the flexural property of the glass/PU composites due to excessive crosslinking that resulted in the composite becoming brittle.
- An increase in the hot-pressing pressure resulted in a reduction in the void content and an increase in the flexural properties of the glass/PU composites.
- Post-curing at 80 °C for 24 h increased the flexural strength and flexural modulus by 42% and 85%, respectively.
- The flexural properties of the glass/PU composites increased with increasing the polymeric binder content. Flexural strength and flexural modulus, respectively, increased by 624% and 273% when the PU content was increased from 5 wt.% to 12 wt.%. The improvement in the flexural properties was attributed to the improved interfacial bonding between the glass particles and the polymeric matrix as the binder content increased. Similar trends were revealed for the tensile and compression properties of the glass/PU composites with increased PU content.
- Improvements in the mechanical properties of glass PU composites manufactured using the optimised hot-pressing conditions and then post-cured were attributed to reduced void content and enhanced glass–polymer interfacial bonding strength.
- The incorporation of glass particles into the PU matrix lowered both the onset and peak decomposition temperatures for the Glass/PU-90/10 and Glass/PU-88/12 composites (see Figure 11(b2)). High loading of glass particles can disrupt the long-range ordering of PU chains as well as reduce the molecular weight of the PU matrix, thereby lowering the onset temperature of thermal decomposition [37].
- No mechanistic changes in the thermal decomposition behaviour of glass/PU were observed when compared to neat PU. The residual char following high-temperature (850 °C) exposure of glass/PU composites in an oxidative environment primarily comprising the thermally inert glass particles.

This research has demonstrated the feasibility of creating glass/PU composites from recovered waste glass. Upcycling waste glass into glass/polymer composites offers an avenue for diverting waste from landfills and the environment through the creation of sustainable and valuable products that have potential applications in the building and construction industry.

## Figures and Tables

**Table 1:** Composition and hot-pressing conditions (e.g., applied pressure, hot-pressing temperature and duration) used for manufacturing the glass/PU composites.

Variables	Glass/PU Composite System	PU Content (wt.%)	Glass Fillers (wt.%)	Pressing Pressure (MPa)	Pressing Temperature (°C)	Pressing Time (min)
Temperature		5	95	6.6	100	1
		5	95	6.6	120	1
	Glass/PU-95/5	5	95	6.6	140	1
		5	95	6.6	160	1
		5	95	6.6	180	1
Time		5	95	6.6	160	1
		5	95	6.6	160	3
	Glass/PU-95/5	5	95	6.6	160	5
		5	95	6.6	160	10
		5	95	6.6	160	30
Pressure		12	88	1.1	160	1
	Glass/PU-88/12	12	88	3.3	160	1
		12	88	6.6	160	1

**Table 2:** Composition and the effect of PU content on the flexural, tensile and compression properties of glass/PU composites manufactured following the optimised process.

Glass/PU Composite System	PU Content (wt.%)	Glass Fillers (wt.%)	Pressing Pressure (MPa)	Pressing Temperature (°C)	Pressing Time (min)
Glass/PU-95/5	5	95	6.6	160	1
Glass/PU-90/10	10	90	6.6	160	1
Glass/PU-88/12	12	88	6.6	160	1

## Data Availability

The original contributions presented in this study are included in the article. Further inquiries can be directed to the corresponding authors.
